# Analysis of Failure Mechanism and Reliability Enhancement of Silicon Strain Gauge-Based Pressure Sensor for Automotive Applications

**DOI:** 10.3390/s24030975

**Published:** 2024-02-02

**Authors:** Hyunchul Sagong, Seongcheol Jeong, Hojoon Lee

**Affiliations:** Reliability Technology R&D Department, Korea Automotive Technology Institute, Cheonan 31214, Republic of Korea; hcsagong@katech.re.kr (H.S.); scjeong@katech.re.kr (S.J.)

**Keywords:** electronic circuits, hydrogen pressure sensor, physics of failure, reliability, silicon strain gauges

## Abstract

Hydrogen fuel cell vehicles have gained more attention as future automobiles due to their environmental benefits and extended driving ranges. Concurrently, the global hydrogen sensor market is also experiencing substantial growth. These sensors are integrated into vehicles to detect hydrogen leakage and concentration, thereby ensuring the safety of hydrogen fuel cell vehicles. In particular, hydrogen pressure sensors, commonly installed on the manifold and regulator of vehicles, can measure hydrogen pressure and diagnose safety concerns caused by hydrogen leakage in advance. In this paper, we identify the vulnerable points of hydrogen pressure sensors when exposed to vehicle driving environments, investigate failure mechanisms, and provide process optimization techniques. Specifically, our reliability modeling verifies that the components of a printed circuit board (PCB) exposed to humid environments undergo corrosion due to ion migration, leading to the generation of extrinsic series or parallel resistances, which in turn cause fluctuations of output voltage. Through structural and elemental analysis, we pinpoint process-related factors that make components vulnerable to humidity, thereby suggesting recommendations for enhancing the manufacturing process. Based on this analysis in the development stage, we can proactively address and improve reliability and further safety-related issues for future automobiles, thus preventing real field issues.

## 1. Introduction

Fuel cell vehicles represent a promising future for the automotive industry that uses the power of hydrogen as a fuel. Through a chemical reaction between hydrogen and oxygen, a fuel cell generates electricity, propelling the vehicle forward. It offers environmental benefits that are similar to electric vehicles while also having the advantage of quick refueling times and extended driving ranges.

Hydrogen is the first element in the periodic table and a promising candidate for future mobility and energy supply [1]. Hydrogen gas comprises two hydrogen atoms united in a molecule. Being the smallest element, hydrogen has a low boiling point, existing as a gas at room temperature and pressure. Due to its low minimum ignition energy, it is prone to easy explosions, and its flame propagation speed is too fast, causing ignition or explosions to easily occur. Furthermore, with a lower flammability limit (LFL) of 4%, it can easily explode with even minimal static electricity and, due to its brittleness properties, it can penetrate metals, forming pin holes or cracks which can weaken vulnerable parts such as vessel and pipeline joints. Safe hydrogen production, storage, and transportation technologies are needed due to these characteristics. The key to prevent and control these aspects lies in the development of hydrogen safety systems with hydrogen sensors, whereas the global hydrogen sensor market is continuously growing along with environmentally friendly and carbon-neutral policies.

The use of hydrogen sensors in vehicles includes various applications such as near hydrogen storage containers, around hydrogen transfer pipelines, adjacent to fuel cell stacks, and within the vehicle cabin. These sensors require a wide detection range, high selectivity, long lifetime, high accuracy, rapid response time, low power consumption, low operating temperature, mass production capability, compact size, and low price. While electrochemical and catalytic technologies have matured over the last few decades and are continuously being improved, semiconductor-based, metal resistance-based, optical-based, and acoustic-based sensor technologies have recently been actively developed [2,3,4,5,6,7,8,9,10,11,12].

In this paper, we focus on resistance-based hydrogen pressure sensors which are commonly installed on the manifold or regulator in hydrogen cars in order to measure the hydrogen pressure. These types of sensors exhibit certain advantages in terms of a very wide detection range and rapid response; however, they have an intrinsic weakness in terms of selectivity [4]. By conducting a comprehensive analysis of processes, reliability, and safety, we demonstrate the failure mechanism of sensors under vehicle driving conditions and provide further process optimization.

## 2. Experimental

To investigate the failure mechanisms, including environmental and structural factors that induce the output voltage fluctuations of the sensor, reliability testing and structural analysis tools were utilized. The environmental factors to which the sensor is mainly exposed are defined as temperature, humidity, and vibration considering the actual driving environments. To assess the impact of these environmental factors, reliability tests were conducted using a temperature cycle chamber, temperature and humidity chamber, and a combined vibration test system.

The temperature cycle chamber allows for an environmental evaluation under conditions of rapid temperature changes, with a temperature range of −70 °C to 180 °C and a heating rate of 15 °C/min. The temperature and humidity chamber enables an assessment of the internal moisture ingress in sealed components under temperature and humidity variations, with a temperature range of −50 °C to 150 °C and a relative humidity range of 25% to 98%. The combined vibration test system allows for the simultaneous testing of vibration in the *x*, *y*, and *z*-axis directions along with temperature cycling.

Through these three evaluations, it is possible to determine which factors among temperature, humidity, and vibration have a significant impact on the sensor’s output voltage fluctuation, which is the focus of this study. For example, by utilizing the results from the temperature cycle chamber and the temperature and humidity chamber evaluations separately, we can decouple the effects of temperature and humidity.

Furthermore, structural analysis tools were utilized to analyze the fundamental structure of the sensor and the structural defects. A three-dimensional X-ray CT inspection system was used to detect defects through high-resolution micro-level X-ray images. The non-destructive visual analysis of features such as voids, cracks, and disconnections on the PCB was mainly performed. SEM-based EDS analysis was performed to observe the surface structure and particle shapes, as well as to conduct compositional analysis of the elements involved. An in situ focused ion beam system with an electron beam resolution of less than 1.0 nm was used for destructive analysis, including deep structure analysis, and the three-dimensional analysis of the sensor.

## 3. Results and Discussion

### 3.1. Structural Anaylsis

**Figure 1:**
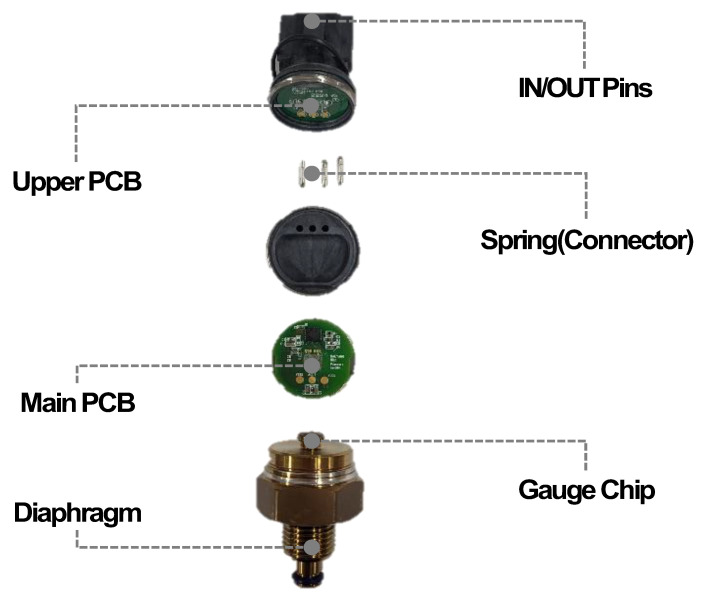
Disassembly analysis of a hydrogen pressure sensor indicates that it consists of components such as diaphragm, gauge chip, main PCB, spring, upper PCB, and IN/OUT pins.

Before the reliability evaluations, a fundamental disassembly analysis was carried out, and the functions of the components comprising the sensor were verified. As seen in Figure 1, the sensor is primarily composed of diaphragm, gauge chip, main PCB, spring, upper PCB, and IN/OUT pins. The diaphragm serves as the inlet for receiving hydrogen pressure, while the gauge chip, in response to the hydrogen pressure (or strain), transmits the resistance change as an output signal to the main PCB. The main PCB, through a signal processing IC, compensates for the electrical input and transmits it as *V_OUT_*. The spring transfers the electrical signal from the main PCB to the upper PCB, resulting in the output voltage through the IN/OUT pins.

**Figure 2:**
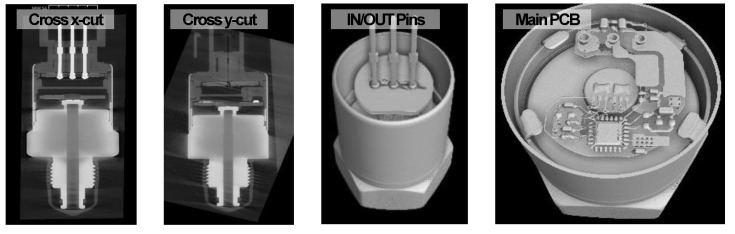

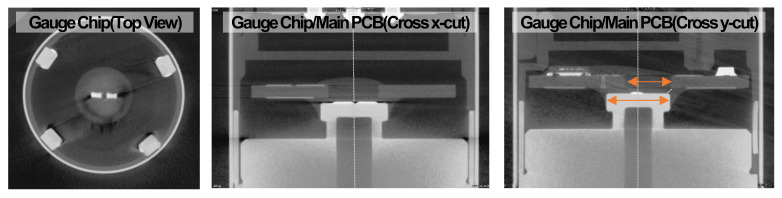
The structural analysis results of three-dimensional X-ray CT indicate that there are no structural abnormalities except for the intentional misalignment.

Figure 2 represents the results of an analysis conducted using a three-dimensional X-ray CT inspection system to identify structural defects or points of weakness. Aside from an intentional misalignment in the area where the gauge chip on diaphragm connects to the main PCB, no significant findings or unusual structural issues were discovered in the detailed structural analysis results.

**Figure 3:**
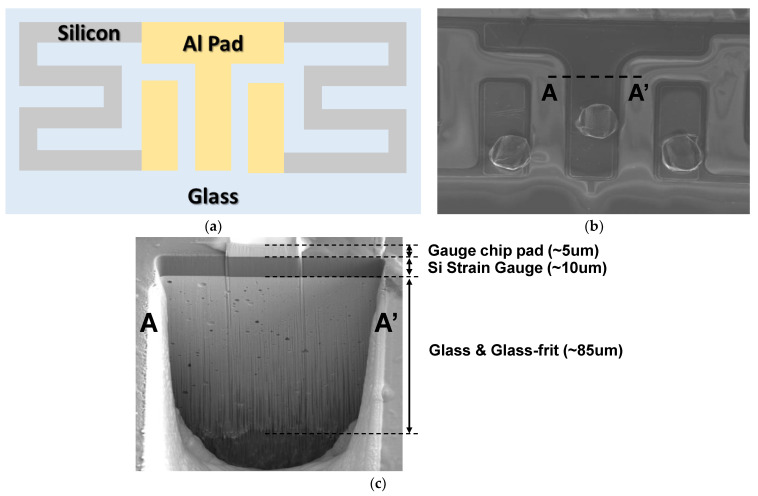
(**a**) Top-view schematic of the gauge chip, (**b**) SEM image of the gauge chip, and (**c**) FIB cross-section image of the gauge chip.

The Si strain gauge chip is fabricated by depositing aluminum onto a silicon–glass wafer, followed by a patterning and etching processes to create the pads. It is composed of two silicon gauges and three aluminum bonding pads arranged on a glass substrate as shown in Figure 3a. The top-view SEM image in Figure 3b confirms the structure. The cross-sectional analysis of the silicon strain gauge using the in situ focused ion beam system shows that it is composed of multiple layers, as illustrated in Figure 3c. These layers include glass frit, the Si strain gauge itself, and the gauge chip pad. The mismatch in the coefficient of thermal expansion (CTE) among these multiple layers can lead to thermal residual stress as a result of temperature changes. The thermal stress can potentially cause serious issues such as Si chip cracking and debonding from the metal diaphragm [13]. To assess the impact of these thermal effects, reliability evaluation and verification follow in the measurement section.

### 3.2. Functional Anaylsis

**Figure 4:**
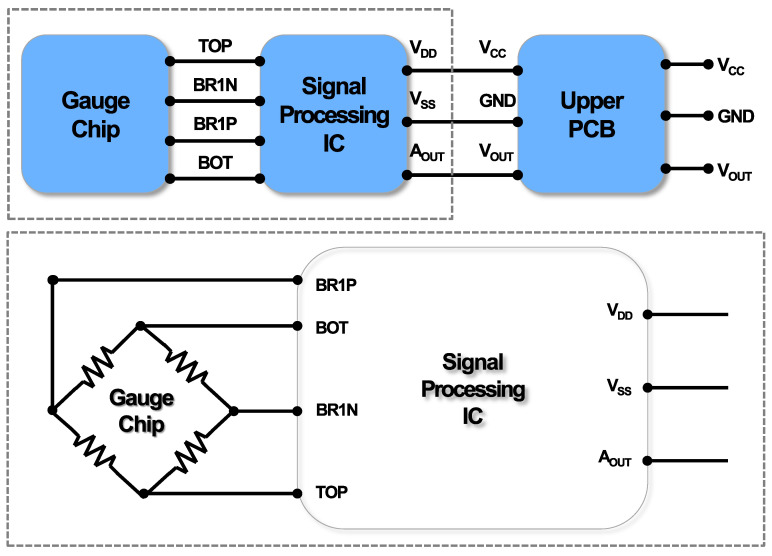
Block diagram through teardown analysis includes the gauge chip, signal processing IC, and upper PCB with connections.

**Figure 5:**
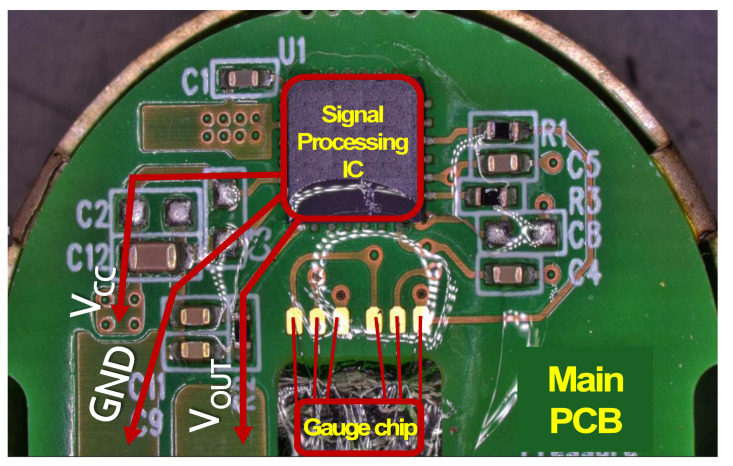
Optical microscope results of the main PCB.

Through a teardown analysis of the sensor, a functional analysis was conducted. The Wheatstone bridge-type gauge chip senses input hydrogen pressure as a resistance change, which is then transmitted to the signal processing IC as an input signal as shown in Figure 4. The output value (*A_OUT_*) processed through the IC is transmitted as the *V_OUT_* signal to external pins via a spring. Power is also connected to the pins. In Figure 5, the optical microscope results for the main PCB indicate the functional connection of each component.

### 3.3. Measurement

The main environmental factors affecting the output voltage fluctuation of the sensor are defined as temperature, humidity, and vibration, taking into consideration actual driving conditions, and structural analysis. The sensor’s potential weak points related to these environmental factors can also be defined as follows:

The first is the bonding areas within the main PCB, including the signal processing IC and parasitic components. In cases where a conformal coating is not uniformly applied across the entire PCB surface, susceptibility to corrosion induced by moisture ingress is increased. The corrosion can lead to unexpected additional IR drop, causing voltage fluctuations in the sensor’s output.

The second point is the solder/wire connections between the gauge chip and the main PCB. The stress induced by vibrations and temperature experienced in real-world conditions can lead to cracking or disconnection, resulting in output voltage variations.

Lastly, there exists a vulnerability from the multi-layer structure of the gauge chip. When subjected to thermal stress, the various material deposition layers within the gauge chip can experience cracking or debonding between the multi-layer structure and the diaphragm due to the mismatch in the coefficient of thermal expansion (CTE) between its multiple layers. It can also lead to output fluctuations and malfunctions in the sensor.

To validate these three weak points, we performed corresponding reliability tests to identify failure mechanisms. First, in order to elucidate corrosion vulnerabilities, we conducted an evaluation under the combined cycling conditions of high and low temperatures as well as high humidity using the temperature and humidity chamber. For the solder/wire cracking and disconnection possibility, we conducted evaluations using a combined vibration test system under temperature cycling conditions, simulating real-world driving vibrations along three axes. Lastly, for the gauge chip, a temperature cycling measurement was performed using a temperature cycle chamber, swiftly transitioning between high and low temperatures at a rate of 15 °C/min. All evaluation conditions comply with ISO 16750 [14].

**Figure 6:**
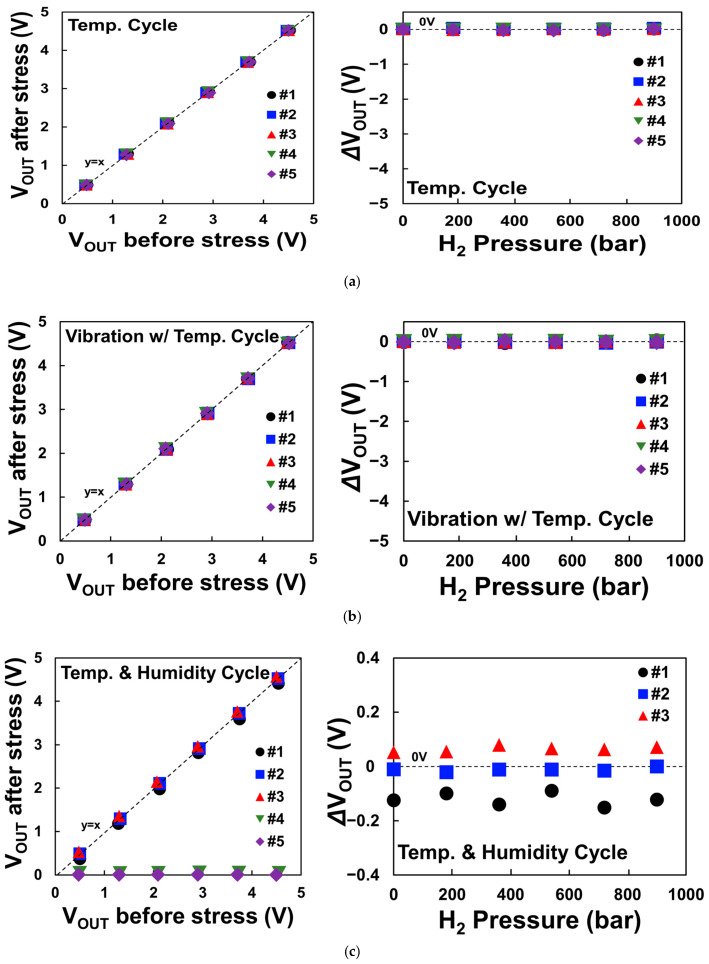
Comparison of output voltage before and after stress due to (**a**) temperature cycling, (**b**) vibration with temperature cycling, and (**c**) temperature and humidity cycling. Δ*V_OUT_* is the result of subtracting *V_OUT_* before stress from *V_OUT_* after stress.

We conducted the three potential points-related reliability tests and measured the output voltage for each sample before and after the stress. Through a sample-to-sample comparison, we can compare the differences in output voltage values before and after the stress and plot the shift value in the output voltage before and after the stress under various pressures. In Figure 6a, it is evident that there is no significant difference in the output voltage before and after the stress due to temperature cycling, indicating a low impact of temperature stress. Similarly, in Figure 6b, the combined vibration test system shows no significant shift in output voltage before and after the stress, also indicating the absence of temperature and vibration effects.

However, during the temperature and humidity test, some samples exhibit an increase, decrease, or even a reduction to zero in output voltage after the stress as shown in Figure 6c. This demonstrates that the hydrogen sensor is susceptible to humidity, as it can either increase or decrease its output voltage following stress. A parametric decrease, such as a decrease in output voltage, is commonly associated with normal degradation behavior. However, the results exhibit an unusual pattern of increase and decrease. In the following section, we present the root cause and the modeling developed to explain this abnormal behavior.

### 3.4. Failure Analysis

**Figure 7:**
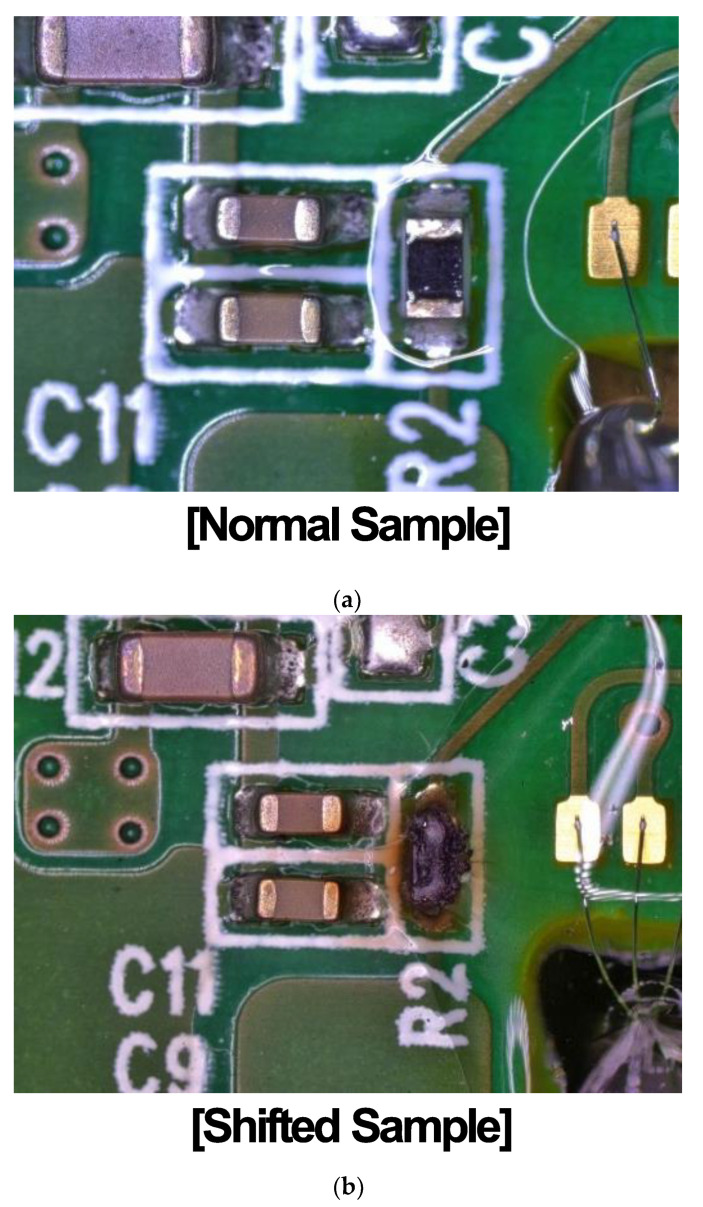
Comparison of main PCB between (**a**) the normal sample and (**b**) the shifted sample.

**Figure 8:**
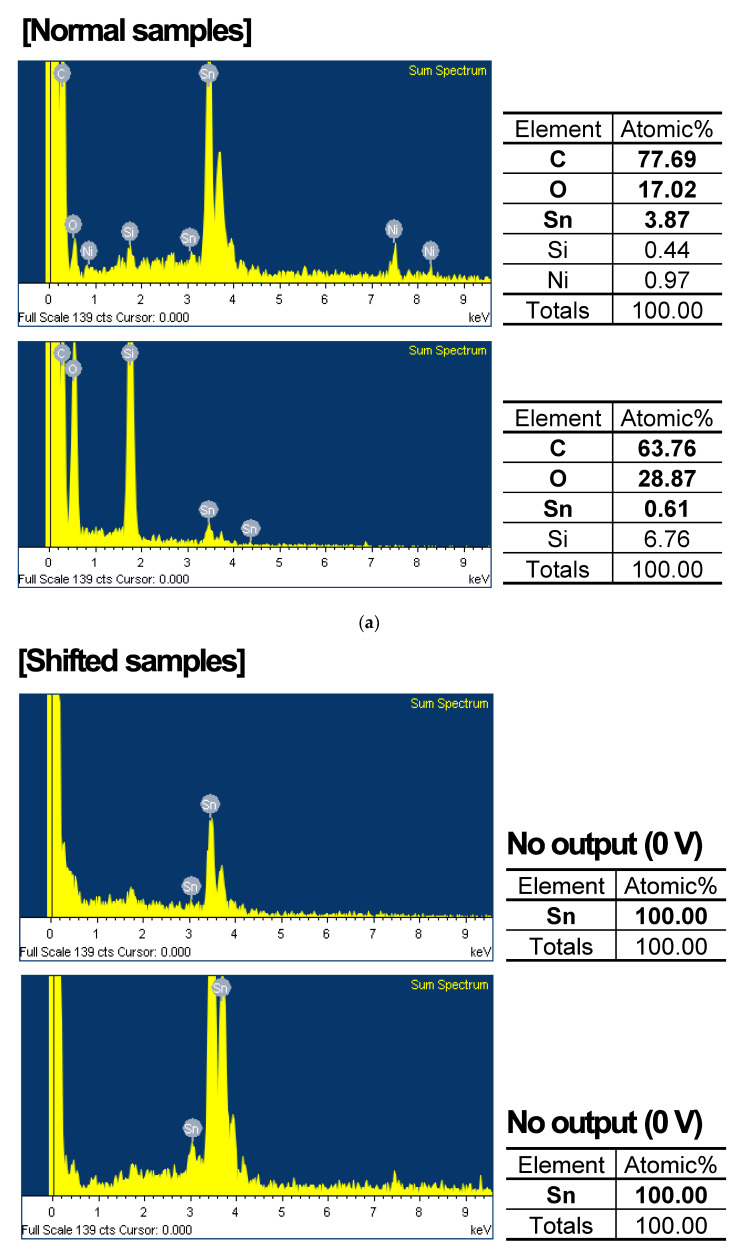

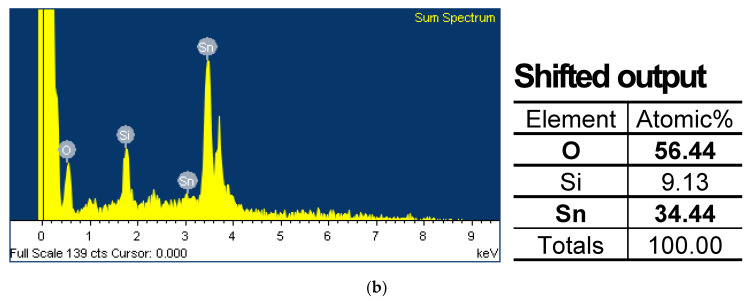
EDS analysis of main PCB of (**a**) normal samples and (**b**) shifted samples after temperature and humidity cycling evaluation.

After the temperature and humidity cycling evaluation, a structural comparison between normal and abnormally shifted samples was carried out using an optical microscope. The normal samples show no noticeable anomalies in the top-view image of the signal processing IC and parasitic components on the main PCB, bonding area, solder/wire, and gauge chip in Figure 7a. However, in the shifted samples, we observed the phenomenon of a specific parasitic component being discolored and corroded while the other area had no significant change as shown in Figure 7b. This can be attributed to the occurrence of thermal and humidity degradation. To identify the root cause and failure mechanism behind the discoloration and corrosion, we conducted a compositional analysis of the element in the affected component using SEM-based EDS analysis, comparing the normal samples and the shifted samples in Figure 8.

In the case of normal samples, the predominant constituents of the conformal coating, such as carbon and oxygen, were detected at levels exceeding 80%. Conversely, these elements were absent or present in significantly lower quantities in the samples with no output and the shifted samples, respectively. In particular, it is confirmed that in the samples where no output values (0 V) were observed, Sn, a metallic component within the PCB substrate, accounted for 100% of the composition. This absence of conformal coating leads to severe malfunction. However, the shifted samples show approximately 50% of the conformal coating components. This analysis indicates that uneven coating is a major factor contributing to the primary defects, resulting in output voltage fluctuation.

### 3.5. Modeling

The reliability test and failure analysis results demonstrate that hydrogen pressure sensors with uneven PCB conformal coatings are vulnerable to humidity, leading to output voltage fluctuations. In the other words, the ingress of moisture into the non-uniform PCB conformal coating results in the temperature and humidity degradation of the exposed circuit. The Arrhenius–Peck model can explain the physical mechanism behind the corrosion phenomenon observed in components which are exposed humidity [15].

(1) $$TTF=A{\left(\%RH\right)}^{-n}\;\cdot \exp\left(\frac{E_{a}}{kT}\right)$$

where *TTF* is time-to-fail, *A* is constant, %*RH* is relative humidity, *n* is humidity factor, *E_a_* is activation energy, *k* is Boltzmann constant, and *T* is temperature.

**Figure 9:**
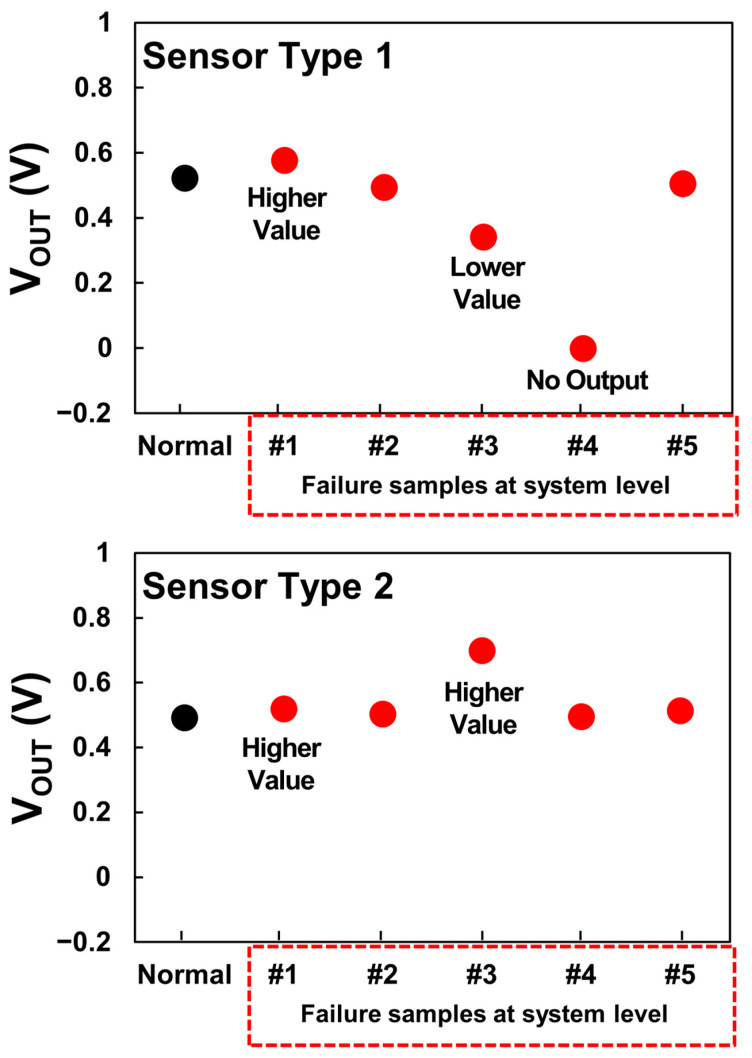
Output voltage characteristics for each failure sample occurring at the system level (including cases of both higher and lower values compared to normal output).

**Figure 10:**
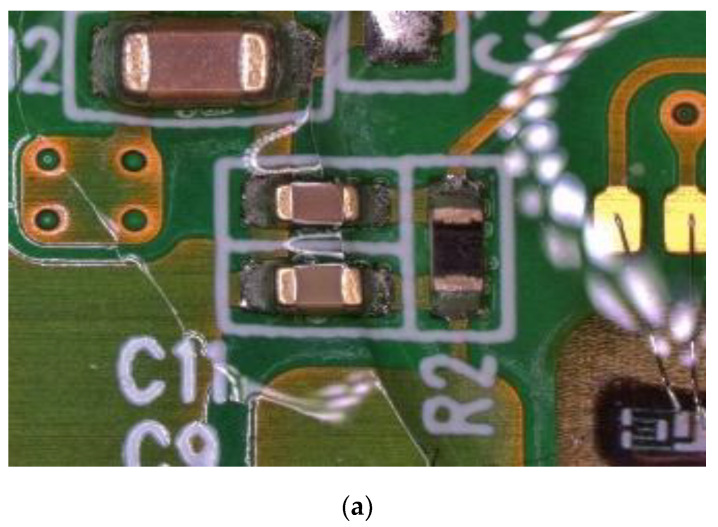

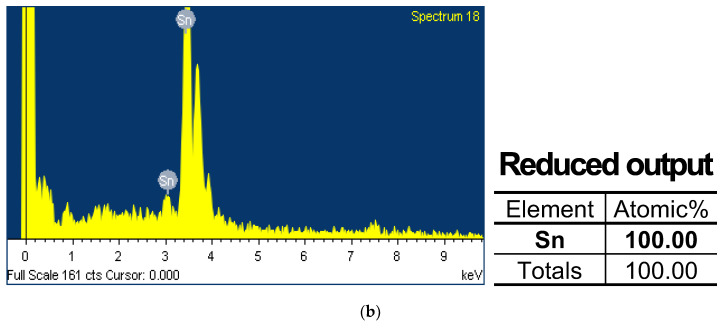
(**a**) Optical microscope image and (**b**) EDS analysis results of the failure sample at system level.

The analysis of the failure samples from system-level evaluation indicates both output degradation as well as increase as shown in Figure 9. The results are aligned with our reliability results from the temperature and humidity cycling test which also demonstrated output voltage fluctuations. This indicates that the reliability test effectively represents a potential reproduction of failures arising from vulnerabilities in the sensor. Figure 10a depicts the observation of the main PCB of a system-level failure sample using an optical microscope, revealing discoloration and corrosion. This corresponds to the outcomes seen in the shifted samples resulting from temperature and humidity cycling. Furthermore, the EDS analysis result shown in Figure 10b also confirms the presence of 100% (Sn) composition, indicating the absence of a conformal coating. These observations suggest a correlation between the absence of conformal coating and the occurrence of system-level failures.

**Figure 11:**
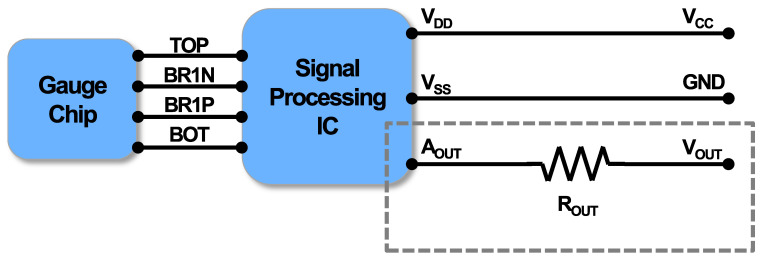

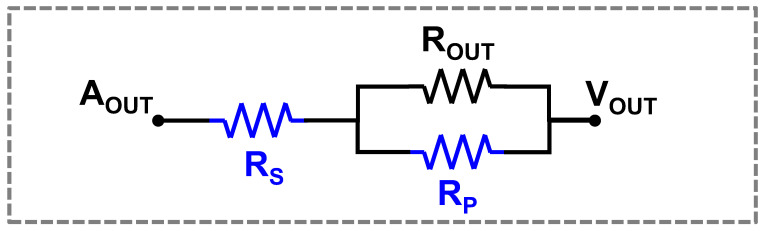
A simple model using humidity-induced extrinsic resistances to explain the output voltage fluctuation.

Consequently, we provided a simple and easy model to explain the voltage fluctuation. After the temperature and humidity cycling test, the discolored and corroded resistor was positioned between *A_OUT_* as the output of signal processing IC and *V_OUT_* and roles as EMC protection and voltage leveling as shown in Figure 11. Once the resistance is exposed to humidity along with its surroundings, extrinsic resistances can be generated due to factors such as ion migration and corrosion [16]. As shown in Figure 11, this extrinsic resistance can be created either in series or in parallel with the resistor. Serial resistance leads to a decrease in output voltage, while parallel resistance results in an increase in output voltage. When serial and parallel resistances are randomly generated, they render the output unpredictable which depends on the amount of both serial and parallel resistance generated for each. This relationship can be expressed using the following simple equation:

(2) $$V_{OUT}=A_{OUT}-\left(R_{OUT}∥R_{P}+R_{S}\right)\times I_{OUT}\;\;\;$$

where *V_OUT_* is output voltage, *A_OUT_* is output of signal processing IC, *R_OUT_* is output resistance, *R_P_* is extrinsic parallel resistance, and *R_S_* is extrinsic series resistance. Table 1 presents the results of case simulation using the versatile model, which effectively predicts both increases and decreases in output voltage. In particular, case 3, as an unexpected case, is presented as an example of an increasing scenario, and a decreasing scenario can also be simulated with a large increase in *R_P_*.

**Figure 12:**
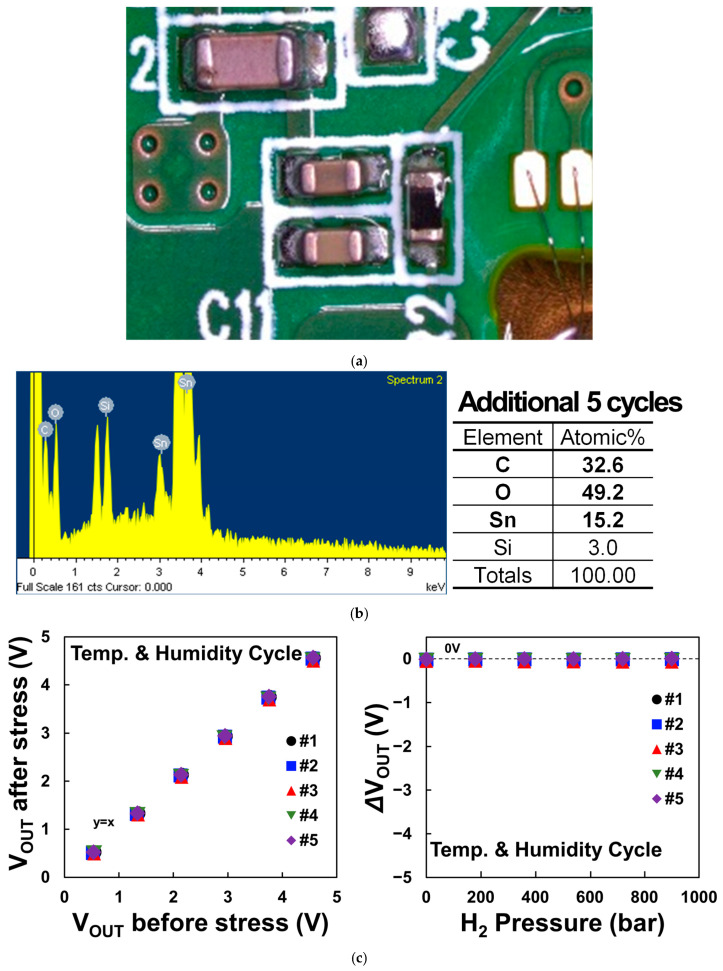
(**a**) Optical microscope image, (**b**) EDS analysis results, and (**c**) comparison of output voltage before and after the additional temperature and humidity cycling stress of the improved samples.

Furthermore, we validated that all failures induced by such uneven conformal coatings can be prevented by improving the uniformity of the coating process and the improved samples show no issues, even in the extended reliability test which includes an additional five cycles of the temperature and humidity cycling test. Figure 12a presents the optical microscope image following an additional five cycles of the evaluation of the improved samples. When compared to the previously observed failure samples, a noticeably reduced level of discoloration and corrosion is evident. Figure 12b illustrates the EDS elemental analysis results, indicating that the composition of conformal coating elements, C and O, stands at 80%, equivalent to the earlier normal samples. Finally, upon comparing the output voltage values before and after the additional 5-cycle temperature and humidity cycling, it is confirmed that they remain at normal levels without any significant increase or decrease in Figure 12c. This observation represents the effectiveness of the improved conformal coating fabrication in enhancing reliability against humidity-induced degradation.

## 4. Conclusions

The comprehensive experimental investigation encompassing both environmental and structural aspects effectively revealed the failure mechanisms and root causes underlying the output voltage fluctuations observed in hydrogen sensors. We specifically identified temperature, humidity, and vibration as the primary environmental factors affecting the sensors, considering actual field driving conditions. Notably, our findings emphasize the substantial impact of humidity on these output voltage fluctuations is significant. 

Structural analysis revealed no abnormalities, except for an intentionally induced misalignment. Additionally, the analysis of the silicon strain gauge’s structure showed potential issues arising from the mismatch in the CTE among its multiple layers from temperature variations. The reliability tests confirmed vulnerabilities associated with the humidity of the sensors. Non-standard output voltage patterns were observed, including both increases and decreases over the conventional reliability models, which also aligns with and is supported by the analysis of failure samples at the system level.

Failure analysis pinpointed uneven PCB conformal coatings as a cause of humidity-induced output fluctuations. The Arrhenius–Peck model can explain the corrosion mechanism caused by humidity and temperature, which effectively provided an explanation for the observed output voltage fluctuations. Moreover, we introduced a simplified and versatile model utilizing humidity-induced extrinsic resistances to understand and predict the output voltage fluctuations. These results will provide valuable insights for enhancing the reliability and performance of hydrogen sensors, ultimately helping prevent field failures in future practical applications.

## Figures and Tables

**Table 1:** Case simulation results.

Parameter	Normal Case	Case 1 *	Case 2 *	Case 3 *
*R_OUT_* (Ω)	22	22	22	22
*R_P_* (Ω)	∞	10	∞	10
*R_S_* (Ω)	0	0	100	10
*A_OUT_* (V)	5	5	5	5
*I_OUT_* (A)	2.0 × 10^−3^	2.0 × 10^−3^	2.0 × 10^−3^	2.0 × 10^−3^
*V_OUT_* (V)	4.96	4.99	4.80	4.97

* Case 1: *V_OUT_* is increased, Case 2: *V_OUT_* is decreased, Case 3: *V_OUT_* is unexpected.

## Data Availability

Data are contained within the article.
